# Prevalence of Surgical Site Infection (SSI) and Its Association With Vitamin D Deficiency

**DOI:** 10.7759/cureus.52015

**Published:** 2024-01-10

**Authors:** Mohammad Eid M Mahfouz, Hanan S Althobaiti, Aryam F Alqthami, Khulud A Alamri, Yousef S Mahfouz, Mahmoud M Elashkar, Maryam M Althomali, Salah Al-Din M Mahfouz

**Affiliations:** 1 Department of Surgery, College of Medicine, Taif University, Taif, SAU; 2 Department of Infection Prevention and Control, King Faisal Medical Complex, Taif, SAU; 3 Department of Endoscopy, King Faisal Medical Complex, Taif, SAU

**Keywords:** s: appendectomy, hemoglobin level, cesarian section, vitamine d deficiency, surgical site infection(ssi)

## Abstract

Background and aim: Surgical site infections (SSIs) are one of the significant complications detected after surgical procedures. Recent studies have highlighted the antimicrobial, wound-healing, and immunological properties of vitamin D. Therefore, this study examined the association between levels of preoperative vitamin D and SSI occurrence in Saudi Arabia.

Methods: We conducted this retrospective observational study among patients who underwent surgery at King Faisal Medical Complex, Saudi Arabia. We included data from patients who underwent surgery between January 2021 and October 2023 in the study. If vitamin D concentrations were not measured at admission, patients were excluded from the final analysis. The researchers performed statistical analysis using the computer program Statistical Package for Social Sciences (SPSS), version 26.0 (IBM Corp., Armonk, NY). The significant level was considered when the p-value was less than 0.05.

Results: The study included 130 patients with a mean (SD) age of 26.98 (9.3) years. Most patients were females (n = 92, 70.8%), had diabetes mellitus disease (n = 121, 93.1%), had a vitamin D deficiency (<30 ng/dl) (n = 106, 81.5%), and underwent cesarean section (n = 80, 61.5%). The mean (SD) vitamin D level among patients was 19.9 (9.7) ng/dl, and the mean (SD) hemoglobin level was almost normal (12.30 (2.1) g/dl). Out of 40.8% (n = 53) of patients, the most detected pathogenic bacteria was *Escherichia coli*, followed by *Staphylococcus aureus* (n = 11, 44%, and n = 7, 25%, respectively). Furthermore, vitamin D deficiency significantly impacted positive SSI; patients with insufficient levels had a higher infection rate compared to those with sufficient levels (n = 58, 54.7% vs. n = 7, 29.2%, p-value = 0.024). A longer surgery duration did not increase the risk of SSI (p-value = 0.047). Patients with class 3 wounds were more prone to SSI than those with class 2 wounds (n = 12, 100% vs. n = 53, 44.9%, p-value<0.001).

Conclusion: This study provides important evidence supporting the relationship between vitamin D deficiency and SSI incidence. Patients with lower levels of vitamin D reported a higher incidence of SSIs. Healthcare providers should pay attention to the high prevalence of vitamin D deficiency among patients undergoing surgery. Screening for vitamin D deficiency and implementing convenient interventions to optimize vitamin D levels could help reduce the incidence of SSIs. Further research with larger sample sizes, more diverse populations, and different surgery types is necessary to validate these findings and explore additional factors influencing SSI development.

## Introduction

Surgical site infection (SSI) is a significant and costly negative outcome of surgical procedures. It also serves as a crucial indicator for assessing the surgeons' and hospitals' performance in quality improvement programs [1-3].

Despite advancements in techniques used in performing surgeries and the widespread prophylactic antibiotics use, SSI remains a prominent issue, with an incidence ranging from 2% to 15% in general surgery. This places it at the first rank of healthcare-associated infections, resulting in increased treatment costs, prolonged hospital stays, and morbidity and mortality [2-6].

The degree of contamination at the surgical site during the operation directly influences the likelihood of SSI development. Wounds are categorized as class I: clean, II: clean-contaminated, III: contaminated, and IV: dirty or infected, depending on the contamination and its degree [7,8].

Microorganisms of either endogenous or exogenous origin can cause SSIs. Most SSIs stem from endogenous microorganisms localized on the patient's skin through the surgical incision, and the causative organisms vary depending on the surgical site. For instance, gastrointestinal tract surgery carries an increased risk of SSIs caused by enteric gram-negative microorganisms. Furthermore, Gram-positive bacteria, particularly *Staphylococcus aureus*, are the most common microorganisms found on the skin and are often responsible for SSIs [9].

Recent studies have paid attention to the significant effects of vitamin D, particularly its antimicrobial properties. Research in the laboratory and among animal models demonstrated vitamin D efficacy in immune regulation, wound healing, cellular growth, intestinal mucosal integrity maintenance, and diabetic ulcers [4,5,10-13].

Nowadays, vitamin D deficiency is regarded as a serious health problem [14]. Regarding the current literature on the Saudi Arabian population, the vitamin D deficiency rate is approximately 60% [15].

While some worldwide studies have investigated the potential impact of vitamin D on nosocomial infections and SSIs, no comprehensive study has yet explored the relationship between vitamin D levels and the development of SSIs in Saudi Arabia [16].

Regarding the widespread prevalence of vitamin D deficiency and its potential implications for SSI, the current study was designed and established to evaluate the relationship between preoperative vitamin D levels and the development of SSI.

## Materials and methods

Ethical approval

The study received ethical approval from the Institutional Review Board (IRB) of the Directorate of Health Affairs in Taif, Saudi Arabia, on September 6, 2023, with IRB approval number HAP-02-T-067.

Subjects

This retrospective observational study was conducted among patients who underwent surgery at King Faisal Medical Complex, Taif, Saudi Arabia. The study included data on patients who underwent surgery in the period between January 2021 and October 2023. Patients whose vitamin D concentrations were not measured at admission or those with incomplete data were excluded from the final analysis. A total of 130 patients were retrospectively collected from the medical records of King Faisal Medical Complex Hospital. The data, such as age, sex, laboratory findings of vitamin D and hemoglobin levels, type and duration of surgery, wound class, the occurrence of SSI post-surgery, and the causative organism, were collected.

Microbiological investigation

Sample collection was carried out by medical officers in the operation rooms, following established departmental guidelines. Specimens’ collection was performed using syringe aspiration or sterile cotton swabs after carefully cleaning the wound with 70% alcohol and then with an iodine solution (1 to 2% tincture of iodine or a 10% solution of Povidone-iodine 1% free iodine). Regarding the superficial wounds, the samples were collected using a 3 to 5 ml syringe with a 22 to 23-gauge needle, aspirating the deepest portion of the lesion. If a vesicle was present, both fluid and cells from the base of the lesion were collected. Regarding deep wounds, the deepest portion of the lesion was aspirated. If the collection was done at the surgery, a portion of the abscess wall was also sent for culture.

After collection, the samples were transported to the microbiology department immediately. Upon arrival in the microbiology department, the swabs were immediately inoculated on various agar plates, including MacConkey agar, chocolate agar, blood agar, and cystine lactose electrolyte-deficient agar (CLED). The plates were then incubated aerobically at 37°C for a period of 24 to 48 hours.

After the incubation period, bacterial colonies that grew on the agar plates were subjected to Gram staining. The Gram-stained bacterial isolates were further referred to biochemical tests for identification and classification.

Statistical methods

The statistical analysis was conducted using the computer program Statistical Package for Social Sciences (SPSS), version 26.0 (IBM Corp., Armonk, NY). Categorical variables were presented in numbers and percentages. A test of normality was conducted for numerical variables. The Chi-square test was done to compare the SSI incidence with categorical variables. The Mann-Whitney test or independent t-test was used to find the association between SSI and numerical variables according to the normality test. The Fisher Exact test was used to find the association between pathogenic bacteria and the wound class. P-values less than 0.05 were considered statistically significant.

## Results

The study included data from 130 patients who had undergone surgery with a mean (SD) age of 26.98 (9.3) years. The majority of subjects were females (n=92, 70.8%) and suffered from diabetes mellitus disease (n=121, 93.1%). The majority of patients (n=106, 81.5%) had a deficiency in the vitamin D level (<30 ng/dl) with a mean (SD) level of 19.9 (9.7) ng/dl. In addition, the mean (SD) of hemoglobin level among patients was normal (12.30 {2.1} g/dl) (Table 1).

**Table 1:** Demographic and clinical characteristics of the patient (N=130). Data has been represented as mean (SD), median (IQR), number, and frequency. SD: Standard deviation; IQR: Interquartile range; N: Number.

Parameters	Category	Value
Age (Years)	Mean (SD)	26.98 (9.3)
	Min-Max	5 – 56
Gender	Male	38 (29.2%)
	Female	92 (70.8%)
Vitamin D (ng/dl)	Mean (SD)	19.9 (9.7)
	Median (IQR)	19.5 (17)
Vitamin D level	Deficiency (<30 ng/dl)	106 (81.5%)
	Sufficient (≥30 ng/dl)	24 (18.5%)
Hemoglobin level	Mean (SD)	12.30 (2.1)
Diabetic	Yes	121 (93.1%)
	No	9 (6.9%)

The majority of patients underwent a cesarean section (n=80, 61.5%). The mean of operation was 54.9±14.8 minutes. After surgery, all patients (n=130,100%) had administrated prophylactic antibiotic therapy, and 90.8% (n=118) had a class II wound type. Regarding SSI, half of the patients did not experience SSI (n=65, 50%), and 40.8% had a superficial infection. The most detected bacterial pathogens among patients who experienced SSI were *Escherichia coli*, followed by *Staphylococcus aureus* (n=11, 44% and n=7, 25%, respectively). Meanwhile, the remaining 40 (61.5%) samples of patients with SSI showed no bacterial growth. The mean duration from surgery to SSI was 9.52±14.4 days (Table 2).

**Table 2:** Surgical procedure and infection-related variables in the study population (N=130). Data has been represented as mean (SD), median (IQR), number, and frequency. IQR: Interquartile range; N: Number; SD: Standard deviation; SSI: Surgical site infection. * Most patients received cefazolin as a prophylactic therapy. **Superficial Surgical Site Infection (SSI): This infection occurs just in the area of the skin where the incision was made. Deep SSI: This infection occurs beneath the incision area in the muscle and the tissues surrounding the muscles. Organ or space SSI: This type of infection can be in any area of the body other than the skin, muscle, and surrounding tissue that was involved in the surgery. This includes a body organ or a space between organs. ***It was found that 2 out of 7 identified Staphylococcus aureus were Methicillin-resistant Staphylococcus aureus (MRSA).

Variable	Category	Value
Type of operation	Appendectomy	50 (38.5%)
	Cesarean section	80 (61.5%)
Duration of operation (min)	Mean (SD)	54.9 (14.8)
	Median (IQR)	53.5 (22)
Wound class	Class II (Clean/contaminated)	118 (90.8%)
	Class III (Contaminated)	12 (9.2%)
Prophylactic antibiotic therapy administration*	Yes	130 (100%)
Type of SSI**	Nil	65 (50%)
	Superficial	53 (40.8%)
	Organ/space	11 (8.5%)
	Deep	1 (0.8%)
Hospital stay from surgery to SSI (Days)	Mean (SD)	9.52 (14.4)
	Median (IQR)	6 (13)
Bacterial growth among patients with SSI	Growth	25 (38.5%)
	No growth	40 (61.5%)
Detected pathogenic bacteria (n=25)	Escherichia coli	11 (44%)
	Staphylococcus aureus***	7 (28%)
	Pseudomonas aeruginosa	3 (12%)
	Mixed bacteria	2 (8%)
	Klebsiella and Pseudomonas	2 (8%)

The most detected pathogenic bacteria in the class 2 wounds were *Pseudomonas aeruginosa* (n=3, 100%), mixed bacteria (n=2, 100%), and *Klebsiella* and *Pseudomonas *(n=2, 100%), followed by *Staphylococcus aureus* (n=6, 85.7%). Regarding class 3 wounds, E. coli was the most frequently detected bacteria (n=4, 36.4%). However, there was no statistically significant association between bacteria type and wound class (p-value= 0.804) (Table 3).

**Table 3:** Association between wound class and detected pathogenic bacteria among SSI patients. Data has been represented as number and frequency. P-values less than 0.05 are considered statistically significant.

Variables		Detected pathogenic bacteria					
		Escherichia coli	Staphylococcus aureus	Pseudomonas aeruginosa	Mixed bacteria	Klebsiella and Pseudomonas	p-value
Wound class	Class 2 (Clean/contaminated)	7 (63.6%)	6 (85.7%)	3 (100%)	2 (100%)	2 (100%)	0.804
	Class 3 (Contaminated)	4 (36.4%)	1 (14.3%)	0 (0%)	0 (0%)	0 (0%)	

The vitamin level significantly impacted positive SSI; a higher positive infection rate was detected among cases with vitamin D deficiency in comparison to those with sufficient levels (n=58, 54.7% vs. n=7, 29.2%, p-value= 0.024) (Table 4).

**Table 4:** Demographic and clinical characteristics of the patients based on surgical site infection. Data has been represented as number and frequency. P-values less than 0.05 are considered statistically significant. a: Independent t-test; b: Chi-square test; IQR: Interquartile range; SD: Standard deviation.

Variables		Surgical site infection		P-value
		Positive	Negative	
Age (years)	Mean (SD)	27.08 (27.1)	26.89 (9.24)	0.910^a^
Sex	Male	21 (55.3%)	17 (44.7%)	0.441^b^
	Female	44 (47.8%)	48 (52.2%)	
Vitamin D level	Deficiency (<30 ng/dl)	58 (54.7%)	48 (45.3%)	0.024^b^
	Sufficient (≥30 ng/dl)	7 (29.2%)	17 (70.8%)	
Hemoglobin level	≤ 12 (g/dl)	25 (44.6%)	31 (55.4%)	0.253^b^
	> 12 (g/dl)	40 (54.8%)	33 (45.2%)	
Diabetic	Yes	2 (22.2%)	7 (77.8%)	0.164^b^
	No	63 (52.1%)	58 (47.9%)	

Operative duration revealed a significant association with the occurrence of SSI favoring the negative SSI; a longer operative duration mean was observed among patients with negative SSI than those with positive SSI (24.14 ±8.84) vs. 15.7 ±8.6, p-value= 0.047). Patients with class 3 wounds were more prone to SSI than those with class 2 wounds (n=12, 100% vs. n=53, 44.9%, p <0.001) (Table 5).

**Table 5:** Effect of different factors related to surgical operation and the incidence of infection. Data has been represented as mean (SD), median (IQR), number and frequency. P-values less than 0.05 are considered statistically significant. a: Chi-square test; b: Mann-Whitney test; IQR: Interquartile range; SD: Standard deviation.

Variables		Surgical site infection		P-value
		Positive	Negative	
Type of operation	Appendectomy	25 (50%)	25 (50%)	1.00^a^
	Cesarean section	40 (50%)	40 (50%)	
Duration of operation	Mean (SD)	15.7 (8.6)	24.14 (8.84)	0.047^b^
	Median (IQR)	15 (13)	25 (12)	
Wound class	Class 2 (Clean/contaminated)	53 (44.9%)	65 (55.1%)	<0.001^a^
	Class 3 (Contaminated)	12 (100%)	0 (0%)	

## Discussion

Surgical infections play a critical role in healthcare due to their association with complications, mortality, and the substantial direct and indirect costs they impose on patients and the healthcare system [1]. Consequently, effective control and prevention of these infections are important. Considering the prevalence of vitamin D deficiency in Saudi Arabia and its relationship to the increased occurrence of SSI, the study’s objective was to explore the association between vitamin D and SSI in Taif, Saudi Arabia.

The assessment of the incidence of SSI holds great importance within quality programs as a crucial indicator for assessing the performance of both surgeons and hospital care [17].

In the present study, the incidence of SSIs among the studied patients was 50%. According to other studies in several countries, the incidence of SSIs was lower. For instance, the SSI incidence in a study in Iran was 9.5%, in the United States was 1.9%, in Italy was 2.6%, in Turkey was 4.1%, and in India was 5%. The variability in the findings may be attributed to the design of the study and surgery types between different studies [12,17-19].

Additionally, the guidelines indicated that dirty wounds have the highest estimation of SSI incidence (over 27%), followed by contaminated (10-17%), and finally, clean/contaminated and clean wounds (3-11% and 1-5%, respectively) [20]. Whereas, in our study, the type of wounds among cases were clean/contaminated and contaminated wounds which may affect the high prevalence of infection.

On the other hand, it was indicated that wound classification and surgical procedure classification as clean, clean/contaminated, or contaminated are significant factors in SSI development [21]. In our study, patients with class 3 wounds (contaminated) were more prone to SSI than those with class 2 wounds (clean/contaminated). Another study in Iran found that a higher incidence of SSIs was observed among patients with clean/ contaminated wounds [17]. In contrast, a study by Abdehgah et al. revealed that there was no significant association between wound class and SSIs [3].

Wound contaminants commonly arise from three primary sources. First, they can originate from the environment, where exogenous microorganisms present in the air or introduced through traumatic injury can enter the wound. Second, the surrounding skin, which harbors normal skin microflora like *Staphylococcus epidermidis*, micrococci, skin diphtheroids, and propionibacteria, can contribute to wound contamination. Lastly, endogenous sources involving mucous membranes, primarily those found in the gastrointestinal, oropharyngeal, and genitourinary tracts, can introduce microorganisms into the wound [22].

In addition, as of the most recent knowledge, there is a prevailing belief among wound care practitioners that aerobic or facultative pathogens, such as *Staphylococcus aureus,* *Pseudomonas aeruginosa*, and beta-hemolytic streptococci, are the main bacteria responsible for delayed healing and infection in both acute and chronic wounds [21].

In the current study, the most detected pathogenic bacteria in the class 2 wounds were *Pseudomonas aeruginosa* and *Klebsiella*, followed by *Staphylococcus aureus*. Regarding class 3 wounds, *Escherichia coli* was the most frequently detected bacteria. Overall, the most detected bacterial pathogens among patients who experienced SSI were *Escherichia coli*, followed by *Staphylococcus aureus*.

According to a similar study, *Staphylococcus aureus* was the most frequently detected organism among patients with SSI, followed by *Pseudomonas aeruginosa* and then *Escherichia coli* [23].

Additionally, bacterial growth was not seen in 61.5% of the samples in our study, which may be attributed to the normal healing process of the wound by the host immune system, appropriate use of antiseptics for cleaning the wounds, or antimicrobial activity. It could also be due to anaerobic bacteria infection, which could be missed as a result of using culture media that only supports the aerobic bacteria [24,25].

Concerning the surgery duration, a previous study found that the prolonged duration of surgery increases the risk of SSI development [24]. Unlikely, our study found that longer surgery duration lowers the risk of SSI incidence. The difference in the findings may be attributed to the type of surgery and quality of care introduced.

Recent research has revealed essential insights into vitamin D effects, such as its antimicrobial properties, immune regulation, promotion of wound healing, cell growth, and positive impact on diabetic wounds [17]. Moreover, evidence provided that both innate and adaptive immune cells possess the vitamin D receptor and exhibit a response to stimulation by 1,25-dihydroxyvitamin D, the active hormone form of vitamin D. This hormone plays a crucial role in the interferon-γ-dependent T-cell response to infections. Additionally, 25(OH)D, another form of vitamin D, connects toll-like receptor activation with innate immunity by increasing the expression of antimicrobial peptides such as cathelicidin (LL-37) and β-defensin. LL-37 has demonstrated potent activity in humans against various pathogens, including bacteria, fungi, viruses, and mycobacteria. It is particularly abundant at barrier sites, suggesting its significance as a primary defense mechanism for the innate immune system [26-29].

On the other side, vitamin D deficiency is considered a major disorder in health systems, with a prevalence ranging from 20 to 80% [30]. Considering a cut-off point of 30 ng/mL in defining vitamin D deficiency, the rate of vitamin D deficiency among our patients was relatively high (81.5%). The high prevalence of vitamin D deficiency is reflected in the high incidence of SSIs. Other studies found a lower prevalence of vitamin D deficiency (39% and 34%) [3,30].

Consequently, our work presents important evidence to support the relationship between vitamin D deficiency and the incidence of SSIs. For instance, vitamin D levels were linked to the incidence of SSIs among our patients; a higher positive infection rate was detected among patients with vitamin D deficiency (54.7%) compared to those with sufficient levels (29.2%). A study conducted in Iran revealed a statistically significant relationship between SSIs and low serum vitamin D levels [17]. Another study by Quraishi et al. revealed that patients with higher levels of pre-operative vitamin D were less likely to develop SSIs [18]. Furthermore, it was found that increased vitamin D deficiency was linked to higher odds of SSI incidence among the patients after the operation [31]. Another study also supported our results and indicated that preoperative vitamin D levels had a strong association with postoperative SSI [3]. These results suggest paying attention to the importance of vitamin D screening among patients undergoing surgery in Taif, Saudi Arabia.

This study is limited to its relatively small sample size, which may limit the generalizability of the results to a larger population. Additionally, the study focused on a specific region (Taif, Saudi Arabia), which may further restrict the generalizability of the results to other geographical areas.

## Conclusions

In conclusion, this study provides important evidence supporting the association between vitamin D deficiency and the incidence of SSIs. Patients with lower levels of vitamin D were found to have a higher incidence of SSIs. Furthermore, the study highlights the significance of wound classification in SSI development, with contaminated wounds showing a higher susceptibility. Screening for vitamin D deficiency and implementing appropriate interventions to optimize vitamin D levels may help reduce the incidence of SSIs.
